# Effectiveness of the cardiac-diabetes transcare program: protocol for a randomised controlled trial

**DOI:** 10.1186/s12913-017-2043-4

**Published:** 2017-02-02

**Authors:** Chiung-Jung (Jo) Wu, John J. Atherton, Richard J. MacIsaac, Mary Courtney, Anne M. Chang, David R. Thompson, Karam Kostner, Andrew I. MacIsaac, Michael d’Emden, Nick Graves, Steven M. McPhail

**Affiliations:** 1 School of Nursing, Midwifery and Paramedicine, Faculty of Health Sciences, Australian Catholic University, 1100 Nudgee Rd, Banyo, Qld 4014 Australia; 2 School of Nursing, Queensland University of Technology, Brisbane, Australia; 3 Royal Brisbane and Women’s Hospital, (RBWH), Brisbane, Australia; 4 Mater Medical Research Institute-University of Queensland (MRI-UQ), Brisbane, Australia; 5 Queensland University of Technology, Brisbane, Australia; 6 Endocrinology and Diabetes, St Vincent’s Hospital Melbourne, Melbourne, Australia; 7 University of Melbourne, Melbourne, Australia; 8 St Vincent’s Institute of Medical Research, Melbourne, Australia; 9 School of Nursing, Director of Centre for Evidence Based Healthy Ageing, Queensland University of Technology, Brisbane, Australia; 10 Director of Centre for the Heart and Mind, Australian Catholic University, Melbourne, Australia; 11 Department of Psychiatry, University of Melbourne, Melbourne, Australia; 12 Department of Epidemiology and Preventive Medicine, Monash University, Melbourne, Australia; 13 Department of Cardiology, Mater Health Services, University of Queensland, Brisbane, Australia; 14 Department of Cardiology, St Vincent’s Hospital Melbourne, University of Melbourne, Melbourne, Australia; 15 School of Public Health & Social Work, Queensland University of Technology, Brisbane, Australia; 16 Australian Centre for Health Services Innovation, Brisbane, Australia; 17 Centre for Functioning and Health Research, Metro South Health, Brisbane, Australia

**Keywords:** Cardiovascular, Diabetes, Randomized controlled trial, Self-efficacy, Protocol, Readmission, Transitional care

## Abstract

**Background:**

This paper presents a protocol for a randomised controlled trial of the Cardiac-Diabetes Transcare program which is a transitional care, multi-modal self-management program for patients with acute coronary syndrome comorbid with type 2 diabetes. Prior research has indicated people hospitalised with dual cardiac and diabetes diagnoses are at an elevated risk of hospital readmissions, morbidity and mortality. The primary aim of this study is to evaluate the effectiveness (and cost-effectiveness) of a Cardiac-Diabetes Transcare intervention program on 6-month readmission rate in comparison to usual care.

**Methods/Design:**

A two-armed, randomised controlled trial with blinded outcome assessment will be conducted to evaluate the comparative effectiveness of two modes of care, including a Usual Care Group and a Cardiac-Diabetes Transcare Intervention (in addition to usual care) Group. The primary outcome is 6-month readmission rate, although a range of secondary outcomes will be collected (including self-efficacy) at baseline, 1, 3 and 6 month reassessments. The intervention group will receive in-hospital education tailored for people recovering from an acute coronary syndrome-related hospital admission who have comorbid diabetes, and they will also receive home visits and telephone follow-up by a trained Research Nurse to reinforce and facilitate disease-management-related behaviour change. Both groups will receive usual care interventions offered or referred from participating hospital facilities. A sample size of 432 participants from participating hospitals in the Australian states of Queensland and Victoria will be recruited for 90% power based on the most conservative scenarios modelled for sample size estimates.

**Discussion:**

The study outlined in this protocol will provide valuable insight into the effectiveness of a transitional care intervention targeted for people admitted to hospital with cardiac-related presentations commencing in the inpatient hospital setting and transition to the home environment. The purpose of theory-based intervention comprising face-to-face sessions and telephone follow up for patients with acute coronary syndrome and type 2 diabetes is to increase self-efficacy to enhance self-management behaviours and thus improve health outcomes and reduce hospital readmissions.

**Trial registration:**

This study has been registered with the Australian New Zealand Clinical Trials Registry dated 16/12/2014: ACTRN12614001317684.

## Background

Cardiovascular disease and diabetes mellitus are contributing to a dual chronic disease epidemic. The Organisation for Economic Co-operation and Development (OECD) reported data compiled from its member’s countries in 2011, identifying that these diseases caused nearly one-third of all recorded deaths [1]. Estimating numbers of patients with both conditions is difficult as data have been primarily collected with single-disease specificity, despite acknowledgement of increasing multi-morbidity [2]. In Australia, where the present study is being conducted, it is difficult to illustrate a complete picture of the proportion of cases of cardiovascular related disease and diabetes, in particular type 2 diabetes mellitus, as there are no national registries of these diseases [3].

Historically, the patient self-management programs that have shown to improve clinical outcomes have focussed on a specific disease or clinical presentation. However, patients with both cardiac conditions and diabetes have higher rates of re-hospitalisation than those with only one disease [4]. A previous study by investigators of this trial identified that patients with both acute coronary syndrome (ACS) and type 2 diabetes were three-fold more likely to be readmitted to an acute hospital within 28 days of discharge, compared to patients with only ACS [4]. Higher readmission rates translate to greater impact physically, emotionally and financially for patients and health services [5].

Inpatient hospital education programs can influence a patient’s management of their chronic disease, yet there is inconclusive evidence on adherence to treatment recommendations for patients with multiple conditions [6]. Qualitative evidence from patients’ perspectives has indicated that disease management programs that only focus on one of an individual’s multiple chronic conditions can create confusion and uncertainty regarding which condition should take priority for treatment [7]. Discharge planning models of care have been identified for patients with singular chronic diseases, and for older patients identified as having risk factors for readmission [8]. A Cochrane Review published in 2013, reported that when discharge planning is tailored to individual needs it is more likely to lead to a reduction in readmission [9]. The present study includes an intervention that can be tailored to the individual needs of patients with both cardiac disease and diabetes with the intention of increasing patients’ self-efficacy in managing both of these conditions.

Programs incorporating strategies promoting self-efficacy have demonstrated potential to translate into behaviour change [10]. The features of the cardiac-diabetes self-management program (CDSMP) to be evaluated in the present study have been discussed in detail previously [11–15]. In summary, the CDSMP has been developed for patients with dual cardiac and diabetes diagnoses and strategies included in the program have their foundation in Bandura’s theory of self-efficacy [16, 17]. Literature examining different program delivery modalities has indicated that a combination of an in-hospital multi-media based education, followed by an early in-home visit and telephone call follow-up can be a relatively inexpensive, interactive and effective approach for reducing unplanned hospital re-admissions [18]. However, this approach has not been trialled specifically among a population of cardiac patients with type 2 diabetes, which is the target clinical group for the Cardiac-Diabetes Transcare intervention to be trialled in this investigation.

### Aims

The aims of the proposed study are to evaluate the effect of the Cardiac-Diabetes Transcare Program on the primary outcome of 6-month readmission rates, as well as secondary outcomes of health status, health-related quality of life, and self-efficacy that will be recorded at baseline, 1, 3, and 6 months. A further aim is to evaluate the cost-effectiveness of this intervention from the perspective of the healthcare system.

## Methods

### Design

**Fig. 1:**
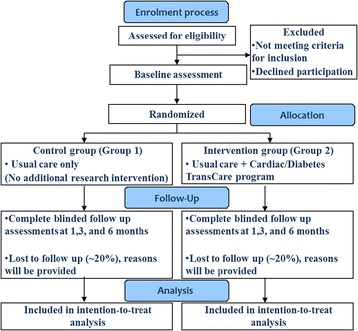
Study design and procedure overview

A two armed, randomised controlled trial with blinded outcome assessment will be conducted with a 6 month follow-up (Fig. 1). This trial will evaluate the comparative effectiveness of Usual Care (Group 1) and the Cardiac-Diabetes Transcare Program (Intervention) which will provide education, in-home visits and telephone follow-up by a Research Nurse (ResN) in addition to their usual care (Group 2).

### Sample size considerations

A target sample of 432 patients (with an anticipated attrition rate of 20% over the 6 month follow-up period) will be recruited from participating hospital facilities in Australia. The participating hospitals include the Royal Brisbane and Women’s Hospital (929-bed tertiary referral public hospital, located in Brisbane, Queensland, Australia), and the St Vincent’s Hospital, Melbourne (504-bed tertiary referral metropolitan hospital, located in Melbourne, Australia).

Completed data on 173 patients per group will enable over 90% power to detect 17.5% difference between groups in 6-month readmission rate (30% versus 47.5%) with alpha = 0.05. The assumptions for this sample size calculation were based on a more conservative anticipated effect than the large effect observed in a subgroup of patients (*n* = 49) with comorbid cardiac condition and diabetes from a randomised trial previously conducted by investigators of the present trial [8]. The previous study similarly included an intervention comprising a home visit and 6-month follow up, but was not tailored for patients with dual ACS and type 2 diabetes and instead targeted older adults who had been hospitalised. The sub-group analysis indicated a large effect of the intervention resulting in a 31.5% (intervention 25.0% versus usual care 56.5%) between group differences in raw 6-month readmission rates.

However, due to potential differences in sample characteristics between the prior trial sub-group and the target sample of the present study that may be associated with readmission rates, an interim analysis of hospital re-presentation event rate will be undertaken. This analysis will examine the 3-month readmission rate in the present study (both groups combined) to see whether it is higher or lower than rates used in the sample size calculation. This will be conducted using the first (approximately) 10% of the target sample to reach the 3-month re-assessment point, by a statistician not involved in the day-to-day operations of the trial. The statistician will be provided with a dataset from each trial site that does not include a variable for group allocation and no attempt to compare event rates between groups during the interim analysis will occur. Rather the sole purpose of the event rate interim analysis is to verify or refute whether the difference in readmission rates used in the initial sample estimate is plausible. If the event rate is substantially discordant with the sample size estimate the investigation team will decide whether an amendment to the trial protocol is warranted, and appropriate approvals (e.g., from ethical review boards) will be sought.

### Recruitment

Patients admitted to study hospitals will only be approached for potential participation if they meet the following inclusion criteria: diagnosed with acute coronary syndrome (ACS) and type 2 diabetes. Acute coronary syndrome is defined as ST-segment elevation myocardial infarction (STEMI), non-STEMI, or unstable angina [19]. Participants will be excluded from participation in the study if they are critically ill, unconscious, on respiratory ventilation, and/or have a current significant cognitive impairment, determined by the treating specialist or his/her nominated physician. Patients eligible for participation will be provided with a participant information and consent form and will be required to provide written informed consent prior to their participation.

Prior to baseline data collection, eligible patients will be recruited by a research assistant (RA) during their hospital admission. The RA will be a hospital staff member and will obtain individual patient’s informed consent. Consenting participants will then be assigned a trial participant number which will be used during the randomisation process where they will be allocated to either the control or intervention group.

### Randomisation, concealment and group allocation

A computerised random number sequence generated by an independent statistician (not involved in the day-to-day trial) will be used to allocate each participant to the control or intervention group in a one-to-one ratio. Group allocation is concealed in opaque envelopes that stored in a lockable filing cabinet accessible to a randomisation gatekeeper (an administration officer not otherwise involved in the conduct of the trial). After a patient has been recruited, the member of the research team responsible for co-ordinating intervention allocation will telephone the randomisation gatekeeper to reveal the group to which that participant has been allocated. Group allocation will then only be known by the randomisation gatekeeper, the person responsible for the intervention allocation and those providing the intervention (ResN) at each participating site. Participants will not be directly told their group allocation (in terms of usual care or intervention). Control group participants will continue to receive the usual standard care while participants in the intervention group will receive usual care as well as the Cardiac-Diabetes Transcare Program.

### Intervention: cardiac-diabetes transcare program

#### While in hospital

After randomisation the ResN visits the participant in hospital and delivers 2 education sessions. These sessions focus on:

1. assessing the patient’s knowledge of their conditions and their skill and confidence in their self-management;
2. providing the patient with examples or models of someone with similar conditions, by showing them relevant segments or the entirety of the digital video of the Cardiac -Diabetes Self-Management Program, “Refocusing Your Life” [12]. The participant will then be given a copy of the digital video (e.g. a Digital Video Disc), a new blood glucose meter and lancing device as well as supplies and blood glucose recording sheets. Opportunities for questions and answers will also be provided during these sessions.

#### After discharge home

The ResN will conduct an in-home visit within the first week post hospital discharge. The visit will ensure participants: 1) have sufficient support and required information; 2) understand individual management goals; 3) are able to carry out self-management activities in the home environment; 4) understand their treatment regimens; and 5) are provided reinforcement and further necessary explanations of self-management.

Following the home-visit, the nurse will conduct weekly telephone follow-ups for 4 weeks, and monthly up to 6 months. The purpose of the individual telephone calls is to ensure participants continue to monitor and manage their conditions, to update their self-management goals according to individual recovery, as well as providing encouragement and feedback to the participant. For patients allocated to the control group, participants will receive usual standard care including routine rehabilitation advice and being referred to a local diabetes educator as the usual treating clinical team deem necessary.

#### Data collection

A RA blinded to group allocation will undertake all data collection. Baseline data will be collected by the RA at the patients’ earliest convenience (within 48 h of recruitment). Follow-up assessments (1, 3 and 6 months) will be conducted at a location convenient for the participant (e.g. at their home) and will be recorded by the RA.

#### Measures

Outcome measures will include: The primary outcome for effectiveness is readmission to hospital, as recorded in patients’ medical record, hospital administrative records, or patient report of readmission to a non-participating hospital (e.g. while travelling). Secondary outcomes include health status (e.g. blood pressure, blood glucose levels documented on the medical record), Assessment of Quality of Life (AQoL) [20] for health-related quality of life, Self-efficacy for Managing Chronic Disease 6-item Scale [21] for self-management, Medical Outcomes Study Social Support Survey [22] for psychosocial well-being, Patient self-report for Health Service Utilisation Questionnaire [23] as well as Medicare and Pharmaceutical Benefits Scheme claims information, and hospital administrative data for healthcare related resource usage to which healthcare costs will be attributed.

### Data analysis

Data analysis will be conducted using intention-to-treat principles. The following analyses will be undertaken:

1. Baseline data for both the intervention and control groups will be examined to check for similarity of the groups. Variables with potential differences between groups at baseline will be controlled for during subsequent analyses.
2. Comparisons between groups will be undertaken to examine the effect of being allocated to the intervention versus control group on the primary and secondary outcomes using generalised linear mixed modelling. These analyses will be adjusted for age and gender in addition to any variables with potential between group differences identified at baseline.
3. Healthcare costs (healthcare system and intervention-related costs) over the 6-month follow-up will be summarised for control and intervention group participants.
4. A trial based economic evaluation will also be conducted alongside the randomised controlled trial (RCT) to quantify the additional costs (or cost savings) per health benefit (Quality Adjusted Life Years (QALYs)) attributable to the intervention in comparison to usual care alone. This will be expressed in the form of an incremental cost effectiveness ratio. All costs will be measured in Australian dollars for the year of study completion. Bootstrap re-sampling of trial data will be used to construct 95% confidence ellipses and cost-effectiveness acceptability curves will be prepared. Sensitivity analyses will be conducted to examine the robustness of these estimates to change in cost, effect, time-horizon, and background context data.

### Ethical considerations

Ethical approvals and agreements have been obtained from all study sites and university settings involved in the study, specifically: Royal Brisbane and Women’s Hospital (Ref No RBWH HREC/14/QRBW/301), St Vincent’s Hospital Melbourne (Ref No: HREC-A 127/4), and Australian Catholic University (Ref No: 2014 309Q) Human Research Ethics Committees, as well as obtaining an approval by the Department of Human Services External Request Evaluation Committee (EREC) (Ref No: MI3435) for accessing healthcare related resource usage and costs from Medicare and Pharmaceutical Benefits Scheme (PBS) database. The trial may be audited according to the practices of the participating facilities at the discretion of their human research ethics committees or research governance offices. All ethical considerations including obtaining individual patient’s informed consent, emphasising voluntary participation, ensuring privacy and confidentiality, secure storage of data, and strict authorised personnel accessibility to the data will be strictly adhered to.

This study does not have a data monitoring committee as it is not a trial of a new drug or medical device. The intervention being trialled is designed to promote adherence to recommended disease management strategies during the transition from hospital to home. It is not anticipated that there will be any adverse events associated with participation in the trial; however, all trial personnel will be encouraged to report any potential adverse events that may occur during the trial through the usual hospital incident reporting systems, as well as to the trial investigators for review and reported directly to the human research ethics committee at the relevant participating site as well as notifying human research ethics committees at other sites.

This study protocol (version 1.01) has been prepared in compliance with the Helsinki Declaration, and has been prospectively registered with the Australia and New Zealand Clinical Trials Registry (ACTRN12614001317684). If any amendment to this trial protocol is required, investigator MC will be responsible for disseminating this information to trial personnel, and human research ethics committees at the participating sites. This study protocol has been prepared following the Standard Protocol Items: Recommendations for Interventional Trials (SPIRIT) guidelines. Trial findings will be reported through peer-reviewed publications with authorship determined in accordance with the International Committee of Medical Journal Editors recommendations.

## Discussion

It has been estimated that approximately 25% of patients with ACS have comorbid type 2 diabetes [24]. These patients have higher morbidity and mortality rates compared to those without diabetes, and have higher rehospitalisation rates [25]. Internationally, the proportion of patients hospitalised with ACS is likely to increase as a result of ageing populations [26] and the number of patients being diagnosed with type 2 diabetes is also rising [1–3, 24]. Higher projected incidence and prevalence of these conditions have made management of these diseases priority areas for research in many countries due to large potential personal and economic costs [1–3, 24, 25].

Evidence from prior research has indicated cardiac rehabilitation and diabetes self-management programs which include strategies for risk factor reduction, increasing physical activity and psychosocial management are beneficial in the management of these conditions and can subsequently decrease hospitalisations [27]. However, there is a paucity of literature reporting the comparative effectiveness of interventions addressing the needs of patients with dual diagnoses of ACS and type 2 diabetes who are transitioning to home after an acute hospital admission. Patients with both conditions may have insufficient adherence to treatment regimens and lower cardiac rehabilitation completion rates [28, 29] that may contribute to negative health events and avoidable hospital presesmntations.

Our previous randomised controlled trials undertaken in a general medical population of hospitalised older adults have demonstrated significant reductions in readmissions, improved quality of life and cost effectiveness through implementation of a theory based intervention comprising home visit and telephone follow up after discharge from hospital [8, 30]. Additionally, our previous work has addressed a number of gaps in the literature regarding needs of ACS patients with type 2 diabetes, and we have piloted cardiac-diabetes self-management programs in preparation for the present trial. Results from this preparatory work have demonstrated the feasibility of the program and a favourable effect on patient self-efficacy [12]. This proposed study builds on previous work through delivering an intervention prepared with a multidisciplinary team, to be evaluated in a randomised controlled trial across two states in Australia to evaluate the clinical and cost effectiveness of this cardiac-diabetes TRANSCARE program.

### Limitations and strengths

This study faces pragmatic challenges associated with investigator initiated trials delivering an intervention at more than one site, alongside administration-related challenges from multi-institutional collaboration. However, this may also be considered a strength of the study as the involvement of more than one study site may enhance the ability to generalise findings from the trial. The investigators have also taken steps to safeguard consistency in the conduct of the trial between sites by ensuring one person is responsible for training the research assistants for the trial, as well as the research nurses delivering the intervention. The same person is also the main contact point for any concerns that may arise during the trial regarding patient recruitment or data collection. Regular monitoring visits will be made throughout the trial to ensure consistency and integrity of the intervention is maintained. Another limitation of the trial pertains to uncertainty that has been described regarding the sample size due to the potential impact of restraints associated with the administration of grant funding, uncertain recruitment rates and no prior effect size data derived from a sample that is closely matched to the anticipated trial population. Instead, power calculations have been estimated for a maximum (target) sample size and possible smaller sample sizes that may occur based on the assumption of a more conservative effect than that observed from a similar intervention approach among hospitalised older adults (not targeted to ACS and diabetes).

## Conclusion

This study protocol has described a randomised trial that will evaluate an intervention program for patients with ACS and Type 2 diabetes commencing in an inpatient hospital setting and transitioning to the home environment. The theory-based intervention comprising face-to-face sessions and telephone follow up aims to increase self-efficacy to enhance self-management behaviours and thus improve health outcomes and reduce hospital readmissions.

## Abbreviations

ACS: Acute coronary syndrome
CDSMP: Cardiac-diabetes self-management program
OECD: The Organisation for Economic Co-operation and Development
RA: Research assistant
RCT: Randomised controlled trial
ResN: Research Nurse
STEMI: ST-segment elevation myocardial infarction.

## References

[1] Organisation for Economic Co-operation and Development OECD (2011). Health at a Glance 2011: OECD Indicators.

[2] Australian Institute of Health and Welfare (2014). Cardiovascular disease, diabetes and chronic kidney disease –Australian facts: Prevalence and incidence. Cardiovascular, diabetes and chronic kidney disease series no. 2. Cat. no. CDK 2.

[3] Tanamas SK. The Australian diabetes, obesity and lifestyle study. In: Baker IDI Heart and Diabetes Institute, editor. 2013.

[4] Wu CJJ, Chang AM (2008). Audit of patients with type 2 diabetes following a critical cardiac event. Int Nurs Rev.

[5] Edmondson D, Green P, Ye S, Halazun HJ, Davidson KW (2014). Psychological stress and 30-day all-cause hospital readmission in acute coronary syndrome patients: An observational cohort study. PLoS One.

[6] Smith SM, Soubhi H, Fortin M, Hudon C, O’Dowd T (2012). Managing patient with multimorbidity: systematic review of interventions in primary care and community settings. BMJ.

[7] Liddy C, Blazkho V, Mill K. Challenges of self-management when living with multiple chronic conditions: Systematic review of the qualitative literature. https://www.ncbi.nlm.nih.gov/pubmed/25642490. PMC4264810 25642490

[8] Courtney M, Edwards H, Chang A, Parker A, Finlayson K, Hamilton K (2009). Fewer emergency readmissions and better quality of life for older adults at risk of hospital readmission: A randomized controlled trial to determine the effectiveness of a 24‐week exercise and telephone follow‐up program. J Am Geriatr Soc.

[9] Shepperd S, Lannin NA, Clemson LM, McCluskey A, Cameron ID, Barras SL (2013). Discharge planning from hospital to home. Cochrane Database Syst Rev.

[10] Sol BGM, van der Graal Y, van Petersen R, Visseren FLJ (2011). The effect of self-efficacy on cardiovascular lifestyle. Eur J Cardiovasc Nurs.

[11] Wu C-JJ, Chang AM, Courtney M, Shortridge-Baggett LM, Kostner K (2011). Development and pilot test of a peer-support based Cardiac-Diabetes Self-Management Program: A study protocol. BMC Health Serv Res.

[12] Jo Wu C-J, Chang AM, Courtney M, Kostner K (2012). Peer supporters for cardiac patients with diabetes: a randomised controlled trial. Int Nurs Rev.

[13] Wu CJJ, Chang AM, Courtney M, Ramis MA (2012). Using user‐friendly telecommunications to improve cardiac and diabetes self‐management programme: a pilot study. J Eval Clin Pract.

[14] Wu CJJ, Chang AM, McDowell J (2009). Innovative self‐management programme for diabetics following coronary care unit admission. Int Nurs Rev.

[15] Jo Wu C-J, Atherton J, Chang AM, Courtney M, Strodl E, Clark A, Thompson DR, Ramis M, McPhail SM (2015). Australian patients using a cardiac-diabetes web-based intervention program: contributions to person-centered clinical practice. Eur J Person Centered Healthc.

[16] Bandura A (1977). Self-efficacy: toward a unifying theory of behavioral change. Psychol Rev.

[17] Bandura A (1997). Self-efficacy: The exercise of control: Macmillan.

[18] Boutwell A, Hwu S (2009). Effective Interventions to reduce rehospitalizations: A survey of the published evidence.

[19] Chew D, Allan R, Aroney C, Sheerin N (2005). National data elements for the clinical management of acute coronary syndromes. Med J Aust.

[20] Hawthorne G, Richardson J, Osborne R (1999). The Assessment of Quality of Life (AQoL) instrument: a psychometric measure of health-related quality of life. Qual Life Res.

[21] Lorig KR, Sobel DS, Ritter PL, Laurent D, Hobbs M (2001). Effect of a self-management program for patients with chronic disease. Eff Clin Pract.

[22] Sherbourne C, Stewart A (1991). The MOS Social Support Survey. Soc Sc Med.

[23] Mortimer D (2008). Protocol for economic evaluation alongside the IMPLEMENT cluster randomised controlled trial. Implement Sci.

[24] Chew DP, French J, Briffa TG (2013). Acute coronary syndrome care across Australia and New Zealand: the SNAPSHOT ACS study. MJA.

[25] Briffa TG, Nedkoff L, Knuiman M, Norman P, Hung J, Hankey G, Thompson P, Geelhoed E, Hickling S, Sanfilippo F, Bremner A, Hobbs M (2013). Downward trend in the prevalence of hospitalisation for atherothrombotic disease. Int J Cardiol.

[26] Smimms AD, Batin PD, Kurian J, Durham N, Gale CP (2012). Acute coronary syndromes: an old age problem. J Geriatr Cardiol.

[27] Panagioti M, Richardson G, Small N, Murray E, Rogers A, Kennedy A, Newman S, Bower P (2014). Self-management support interventions to reduce health care utilisation without compromising outcomes: a systematic review and meta-analysis. BMC Health Serv Res.

[28] Grace SL, Oh PI, Marzolini S, Colella T, Tan Y, Alter DA (2015). Observing temporal trends in cardiac rehabilitation from 1996 to 2010 in Ontario: characteristics of referred patients, programme participation and mortality rates. BMJ Open.

[29] Listerman J, Bittner V, Sanderson BK, Brown TM (2011). Cardiac rehabilitation outcomes: Impact of comorbidities and age. J Cardiopulm Rehabil Prev.

[30] Graves N, Courtney M, Edwards H, Chang A, Parker T, Finlayson K (2009). Cost-effectiveness of an intervention to reduce emergency readmissions to hospital among older patients. PLoS ONE.

